# Supplementary material for: Shared Treg dysregulation underlies psoriasis vulgaris and atopic dermatitis through the PHF19-PRC2 epigenetic axis
Source: Front Immunol. 2026 Aug 20;17:1932788. doi: 10.3389/fimmu.2026.1932788 (PMC13538097; doi:10.3389/fimmu.2026.1932788)
Supplement: Supplementary file 3 [file DataSheet3.zip › Additional files/Additional file 4/28.NIP7.leaveoneout.pdf]

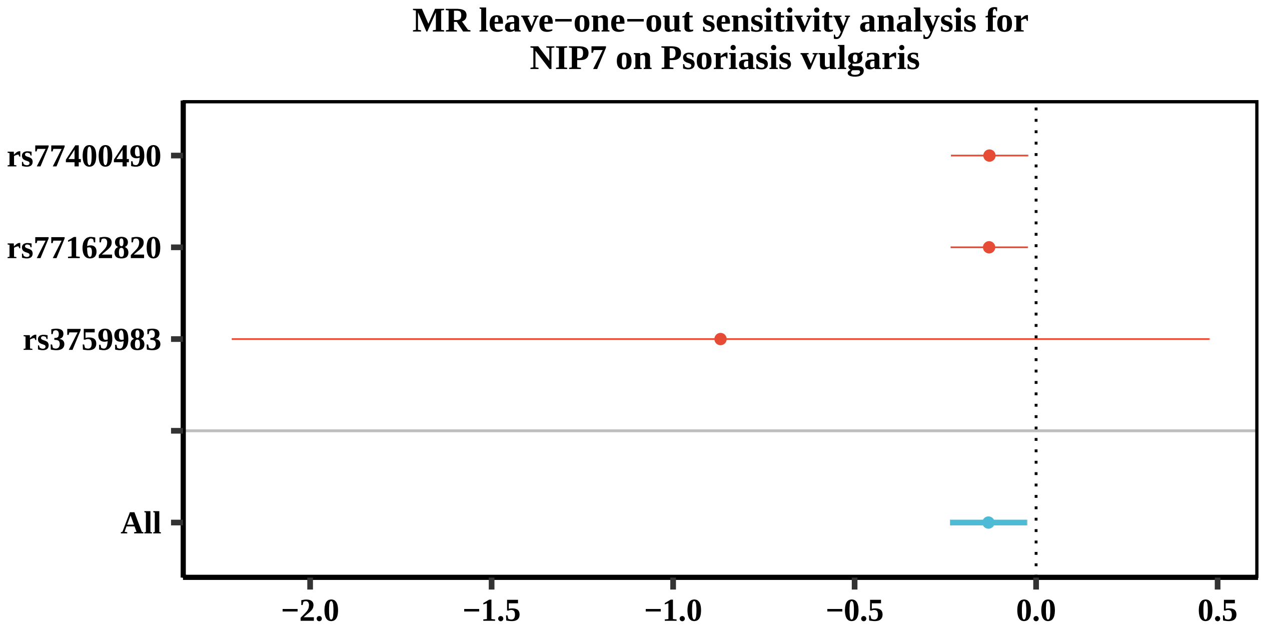
MR leave−one−out sensitivity analysis for
NIP7 on Psoriasis vulgaris
rs77400490
rs77162820
rs3759983
All
−2.0
−1.5
−1.0
−0.5
0.0
0.5
